# The Education Features of the Dental Association

**Published:** 1898-04

**Authors:** J. Percy Corley


ARTICLE VI.

### THE EDUCATION FEATURES OF THE DENTAL ASSOCIATION.

BY J. PERCY CORLEY.

**Abstract of a paper read before the Southern Dental Association.**

The three great educational factors are, the journal, the college, and the Association. Our dental literature has contributed more to the advancement of the profession socially and scientifically, than any other department of the educational *regime*, and as an outgrowth of its influence we have the college and the Association, whose high curricula and chase code of ethics are the source of pride to every son of America.

The college professor is the vessel which bears the inspiring draught to our thirsty lips; he should be, and often is, an artist with instinctive genius for discerning the scattered forces of our nature and arranging them in harmonious accord.

The Association is a great confessional, where we relate our failures, pointing to where the hidden reef lies beneath the glassy surface. We bring our problems and ask advice. We also bring the treasures which we gather as we climb the rugged steeps of experience and scale the mountains of difficulties. The Association is also a great store-house which holds within its capacious vaults the accumulated wealth of hundreds of lives devoted to toil and study. It is a kind of professional Mecca to which we make annual pilgrimages to pay tribute at the shrine of progress, and acquire strength for the ever increasing responsibilities of life.

Upon the shoulders of the dental profession rests the burden of educating the public. There is scarce an institution of learning to-day which has not in its curriculum a treatise on "physiology and hygiene," and its campus and gymnasium for the cultivation and development of the body; but *the oral cavity*, the most important department in the human economy, is passed with scarcely a word. The tooth-brush is more important than dumb-bells, and dental sanitation the first step in hygienic routine. I admit the need of dental surgeons in the army and navy, but the nursery needs dental attention more than the garrison, and the school boy more than the soldier. The sooner we recognize in the boy the fetus of the man, and the baby as our most important patient, the sooner will we attain the ideal in dental achievement.

It has been said that the Association consists of egotists seeking for office; the college professor electioneering for students; the inventor advertising his appliances, and the depot man peddling his wares! But if a man is qualified to serve his profession in an official capacity, it is his duty to use legitimate means for the attainment of such a position of usefulness and honor; it is the duty of the college to let the profession know the material she has in her faculty and to demonstrate the advantage offered; it is kind of the inventor to show us his appliance and show us how to use, thus saving us from investing in something else we might be unable to use to advantage; and the depot man confers a genuine favor on those of us who live in the country, by his magnificent displays of all the paraphernalia of our profession. So these accusations bring no discredit upon the Association. May this convention mark the dawn of a new era in dentistry which shall spread as the morning light, until the dark shadows of ignorance and superstition shall havh given place to the glory of intellectual day. The social, intellectual and clinical features of the "Southern" have stood for years without a parallel. Let us then build upon the prestige of the past a "Parthenon" for the future. The wise Creator did much for every clime; but when the Lord made "Dixie" he did his best.

The ideal dentist is an incarnation of German learning, French art, English thoroughness, American push, and Southern chivalry.—*American Dental Weekly.*

